# Helicobacter pylori: An Underrated Cause of Immune Thrombocytopenic Purpura. A Comprehensive Review

**DOI:** 10.7759/cureus.5551

**Published:** 2019-09-01

**Authors:** Muhammad A Zain, Fahad Zafar, Ammar Ashfaq, Abdur R Jamil, Asrar Ahmad

**Affiliations:** 1 Internal Medicine, Sheikh Zayed Medical College and Hospital, Rahim Yar Khan, PAK; 2 Internal Medicine, Maimonides Medical Center, Brooklyn, USA; 3 Internal Medicine, Abington Hospital - Jefferson Health, Abington, USA; 4 Internal Medicine, Central Michigan University, Saginaw, USA

**Keywords:** h. pylori, immune thrombocytopenic purpura (itp), itp, helicobacter pylori infection, immune thrombocytopenia

## Abstract

Idiopathic thrombocytopenic purpura (ITP) is the autoimmune-mediated destruction of platelets. ITP is a diagnosis of exclusion after other identifiable etiologies have been ruled out. After the first report by Gasbarrini et al. (1998) showing rising platelet counts in ITP patients following *Helicobacter pylori* (HP) eradication therapy, there is growing evidence that highlights the role of HP in triggering ITP. However, the exact pathophysiology of HP-associated ITP is still unclear, but many theories have been implicated in this regard. According to various reports, the postulated mechanisms for the role of HP in cITP include molecular mimicry, increased plasmacytoid dendritic cell numbers, phagocytic perturbation, and variable host immune response to HP virulence factors. One famous theory suggested molecular mimicry between platelet surface antigen and bacterial virulence factor, i.e. cytotoxin-associated gene A (CagA). It is thought that a chronic inflammatory response following an HP infection induces the host autoantibodies' response against CagA, which cross-reacts with platelet surface glycoproteins; therefore, it may accelerate platelet destruction in the host reticuloendothelial system. However, further studies are mandated to better understand the causal link between ITP and HP and study the role of biogeography. Nowadays, it is recommended that every patient with ITP should undergo HP diagnostic testing and triple therapy should be administered in all those candidates who test positive for HP infection. In our review, there were a few pregnant female ITP patients who took HP eradication therapy mainly after 20 weeks of gestation without maternal or fetal worst outcomes. However, large-scale studies are advisable to study the adverse fetal outcomes following triple therapy use.

## Introduction and background

*Helicobacter pylori *(HP) is a spiral-shaped, microaerophilic, gram-negative bacillus, first isolated from a gastric biopsy in 1983 [1]. HP is prevalent in more than half of the world’s population with the majority of individuals, around 80%, infected in developing countries. The prevalence positively depends upon increasing age, poor socioeconomic conditions, population density, smoking, as well as untreated water supplies contaminated with fecal matter from infected individuals [2]. HP can be contracted as early as childhood. This bacteria is transmitted via the fecal-oral or oral-oral route and colonizes the gastric mucosal lining of the infected individuals for a lifetime. The spontaneous eradication of an HP infection rarely happens but is possible with antibiotics taken for an unrelated illness. HP has been implicated in various upper gastrointestinal diseases, including chronic gastritis, gastric atrophy, peptic ulcer disease (PUD), gastric lymphoma (MALToma), and distal gastric adenocarcinoma [3]. HP was declared as a first-grade carcinogen by the International Agency for Research on Cancer (IARC) in 1994 [4]. The virulence factors differ geographically between Western and Eastern Asian HP strains where the Eastern Asian strain is associated with a higher risk of gastritis and gastric cancer. Recently, HP has been linked with many extra-gastric disorders such as pernicious anemia, autoimmune thyroiditis, rheumatoid arthritis, coronary artery disease, and immune thrombocytopenic purpura (ITP) [5-6].

Immune thrombocytopenic purpura (ITP), an autoimmune hematological disorder, is characterized by autoantibodies-mediated platelet destruction in the reticuloendothelial system and/or abnormal maturation of megakaryocytes in the bone marrow [7]. ITP is a diagnosis of exclusion, with fewer than 100000 platelets per liter of blood as the cut-off platelet count for making the ITP diagnosis [8]. ITP is characterized as acute (diagnosis to 3 months), persistent (3-12 months), or chronic (>12 months). ITP in children is of acute onset and predominantly a self-limited disease, with 70% of affected children recovering completely in the first six months even without any treatment. However, ITP in adults starts insidiously and is chronic with a 20%-40% chance of full recovery later on [9]. The average age of ITP onset in adults is 56-60 years [10]. ITP onset can be primary or secondary. Primary ITP has no identifiable underlying etiology whereas secondary ITP has an identifiable causative agent such as genetic susceptibility, the presence of certain environmental factors, neoplastic conditions, or a bacterial or viral infection such as human immunodeficiency virus (HIV), hepatitis C virus (HCV), or chronic HP infection. A chronic inflammatory response may be induced following a recent bacterial or viral infection resulting in the production of host antibodies, which can cross-react with the platelet surface antigen, thereby augmenting accelerated platelet clearance in the host reticuloendothelial system.

## Review

Pathophysiology of H. pylori-induced ITP

In 1951, WJ Herrington was treating a patient with unexplained thrombocytopenia at Washington University [11]. He infused blood from this particular patient to himself and a few healthy volunteers, leading to a drop in their circulating platelet levels. Several years later, immunoglobulin G (IgG) was revealed to be a component of that infused blood leading to antibody-mediated platelet destruction. After the first report by Gasbarrini et al. (1998) showing rising platelet counts in ITP patients following HP eradication therapy, HP was suspected in triggering the secondary ITP. However, the exact mechanism by which HP causes platelet destruction is still unknown.

After ingestion, HP manages to colonize the mucosal lining of the stomach by eluding the host innate immunity through various adaptive mechanisms, including neutralizing the acidic stomach environment by the production of ammonia using urease enzymes, altering the mucus viscosity in order to have easy mobility, motility due to flagella to avoid being washed out of the stomach by peristalsis, anergic lipopolysaccharide (LPS) cell wall/flagella, and having various adhesion proteins to help attach gastric epithelial cells (ECs) [12].

According to various reports, the postulated mechanisms for the role of HP in cITP include molecular mimicry, increased plasmacytoid dendritic cell numbers, phagocytic perturbation, and a variable host immune response to HP virulence factors.

HP have diverse genes for coding the multiple outer membrane proteins (OMPs) and virulence factors. These OMPs, such as blood group antigen-binding adhesion A (BabA), outer inflammatory protein A (OipA), and sialic acid-binding adhesin (SabA), facilitates the binding of HP to gastric ECs [13-15]. Among the various virulence factors, the two most important ones are vacuolating cytotoxin A gene (VacA) and cytotoxin-associated gene A (CagA). The CagA gene is located in the 40kb cluster of terminal genes on the cytotoxin antigen pathogenicity island (Cag PAI), and it codes for a type IV secretion system (T4SS) along with CagA protein. Patients infected with Cag PAI⁺ HP strains are more likely to develop peptic ulcers or gastric cancer due to CagA oncoprotein [16]. The T4SS acts as a vehicle to translocate the CagA protein into the host gastric ECs [17]. Once phosphorylated inside the ECs, CagA protein evokes a host systemic immune response in a host by inducing IL-8, a strong proinflammatory cytokine [16,18-19]. Within gastric ECs, phospho-CagA activates a eukaryotic phosphatase (SHP-2) as well as ERK, a member of the MAPK family, leading to altered ECs signaling and growth factor stimulation.

CagA protein is highly antigenic and induces anti-cagA antibodies. It is suggested that molecular mimicry exists between cagA and platelet-associated IgG (PAIgG). Anti-cagA antibodies (Abs) crossreact with GPIIb/IIIa platelets surface antigen and results in an accelerated immune complex formation and clearance of platelets in the host RES. Another theory suggests the enhanced platelet activation and clearance in the host RES due to the interaction of HP-bound von Willebrand factor (VWF) with platelet surface Ag (GPIb) [20].

On the other hand, monocytes from HP-positive patients demonstrate the low levels of the inhibitory Fc-γ receptor IIB which results in enhanced platelet phagocytosis, thereby, supporting the increased phagocytic perturbation theory. It is further supported by the upregulation of the inhibitory Fc-γ receptor IIB signaling following successful eradication of HP [21].

The second most important virulence factor is VacA, which blocks the proliferation of helper T cells by interfering with the T-cell receptor interleukin 2 (IL-2) pathway. It is thought that the binding of VacA to multimerin-1 on platelets may result in enhanced platelet activation and clearance. 

Diagnosis and treatment

ITP is characterized by a low platelet count and can lead to life-threatening bleeding. Therefore, the diagnosis and evaluation of the underlying etiology are very important for appropriate treatment. Since ITP is a diagnosis of exclusion, appropriate testing must be done to identify all the possible causes of low platelet count. Also, testing needs to be done to exclude infections like HIV and HCV. The reliable non-invasive diagnostic tests for HP testing in ITP patients include urea breath test, serological tests for anti-HP antibodies, and stool antigen tests [22]. The invasive tests include endoscopy with biopsy and urease test performed on the biopsy specimen. Culture and sensitivity are not recommended unless there has been a treatment failure [23].

The traditional treatment of ITP largely includes immunosuppressive agents, such as corticosteroids (prednisone), immunoglobulin therapy (IVIG and anti-D), Rituximab, and salvage splenectomy. These treatment modalities are not only expensive but have multiple side effects [24]. Moreover, about 10%-20% of patients either do not sustain a normal platelet count or develop a relapse despite these expensive treatments [25]. With recent clinical reports showing the emergent correlation between HP and ITP, it is highly suggested to detect and treat the infection. HP eradication therapy includes the combination of antibiotics (clarithromycin, metronidazole, amoxicillin) with proton pump inhibitors. The usual duration of treatment is seven to 14 days. HP stool antigen testing and blood platelet count is done eight weeks post-therapy to check the efficacy of treatment [25]. It has shown promising results with improved platelet counts and the normalization of auto-platelet antibodies without relapse [26]. Also, these treatment modalities have fewer side effects and are cost-effective. Another interesting fact is the correlation of treatment with geographical location, with higher response rates in Japan and Italy (28%-100%) than in the US and other European countries (<13%). This highlights the significance of patient biogeography and regional HP strain variations in the treatment of HP-induced ITP [27-28].

This article emphasizes the strong association between HP and chronic ITP using a comprehensive review of cases reported in the literature. We have used the Bradford Hill Criteria to establish the association between HP and ITP. First, there must be a close association between HP and ITP. Second, there must be some reasonable biological mechanism behind it. Third, the infection should occur before ITP and, eventually, the eradication of infection should eliminate the disease (i.e. increased platelet count) and eradicate the infection (evidenced by a decline in antiplatelet antibodies and negative HP testing) [29-30]. We conducted a structured literature search of PubMed (National Library of Medicine, Bethesda, MD) using a combination of terms, including “chronic ITP” and "*H Pylori *eradication therapy." After carefully reviewing the relevant literature consisting of but not limited to the original articles, case series, and case reports, a total of 12 cases were found that were available in full-text form and met the Bradford Hill criteria [30-36]. The data of individual cases of “*Helicobacter Pylori *induced chronic ITP” on epidemiology, clinical presentation, diagnosis, management, prognosis, and the outcome are also summarized in Table 1.

**Table 1:** Summary of HP-induced ITP published cases meeting Bradford Hill Criteria. HP: Helicobacter Pylori; ITP: immune thrombocytopenic purpura; RX: treatment; F: female; M: male; OD: once daily; BD: twice daily; GI: gastrointestinal; PCR: polymerase chain reaction; PPI: proton pump inhibitors; HBV: hepatitis B virus; HIV: human immunodeficiency virus; EBV: Epstein-Barr virus; CMV: cytomegalovirus; PAIgG: Platelet-associated Immunoglobulin G

Author	Publication year	Country	Age/ Gender	Clinical presentation	Diagnostic investigation findings to identify secondary ITP etiology	Platelet count before Rx	Treatment	Outcome / Platelet count after eradication therapy
Hill et al. [30]	2014	USA	54/F	Asymptomatic, low platelet count was identified incidentally on routine examination	HIV negative HP positive	47000 cells per mL	Initial Rx with prednisone 40 mg OD increased platelet count to 135000 cells/mL in 3 months. The patient then developed refractory thrombocytopenia in next 5 years. After HP test came positive, the patient was treated with triple therapy (Clarithromycin 500 mg BD Amoxicillin 1000 mg BD Lansoprazole 30 mg BD) for 14 days.	After RX, platelet count rose to 145000 cells/mL (highest in 15 years) and remained stable. Stool antigen test 1 year later was negative for HP
Tiwari et al. [31]	2009	India	40/F	Bleeding gums, generalized purpura, bleeding into the right eye, Malena, abdominal pain, and vomiting.	Upper GI endoscopy: Fundal and corpus hemorrhagic gastritis. Biopsy for HP came positive.	40000 cells per mL	Initial resuscitation followed by triple therapy and steroids	After 3 months of treatment, endoscopy and PCR was negative for HP and platelet count was normalized
Kobayashi et al. [32]	2014	Japan	78/M	Referred to as a case of progressive thrombocytopenia	Anti HP IgG positive	51000 cells per mL	Triple therapy with amoxicillin, clarithromycin, and PPI	Platelet count after 2 weeks of therapy was 88000 cells/mL. However, a urea breath test was positive and the patient developed sec HP infection. Retreatment with triple therapy resolved the infection with a negative HP breath test and sustained higher platelet count of 125000 cells/mL than baseline
Arend et al. [33] (Case 1)	2012	Netherland	75/M	Mild epistaxis and ecchymosis	Urea breath test positive	7000 cells per mL.	Treatment with high dose steroids and immunoglobulins were unsuccessful. Splenectomy was contraindicated due to a recent aortic prosthesis. Due to a positive urea breath test, HP triple therapy was given.	Platelet count increased to 140000 cells/mL in 4 months. Urea breath test came negative for HP
Samson et al. (Case 2) [33]	2012	Netherland	44/M	Admitted with renal colic and an incidental diagnosis of ITP.	HBV, HIV, EBV, CMV serology negative. Bone marrow tests and imaging of the abdomen and thorax were normal. Urea breath test positive for HP	15000 cells per mL	Triple therapy was given. Platelet count increased to 100000 cells/mL in 1.5 months After few months platelet count fell again to 51000 cells/mL with a positive urea breath test for HP. Quadruple therapy was given.	Platelet count normalized to 125000 cells/mL within 5 months of quadruple therapy and remained stable. Also, the urea breath test was negative
Etou et al. [34]	2013	Japan	41/F	Hematochezia owing to UC, with incidental finding of thrombocytopenia.	Endoscopy showing mild proctitis as the patient was a diagnosed case of UC for over 10 years. WBCs and RBCs normal. Pt, aPTT, fibrinogen and D dimers all normal. Platelet-associated IgG was 107 pg/ml (normal is 0-46). Urea breath test came positive and upper GI endoscopy showed atrophic gastritis	10000 cells per mL	Drug-induced thrombocytopenia was suspected initially but stopping Mesalamine didn’t improve platelet count. Since HP testing was positive, so triple therapy was given with significant improvement of platelet counts.	Platelet counts improved after triple therapy and remained stable thereafter.
Ono et al. [35] (Case 1)	2017	Japan	31/F G1P1	Incidental low platelet count during pregnancy	Platelet count of 5600 at 13 weeks and 3500 at 21 weeks of gestation. ITP diagnosed after excluding other causes. Bone marrow aspiration denied. Urea breath test positive	3500 cells per mL	HP eradication therapy (i.e. amoxicillin 750 mg BD, clarithromycin 400 mg BD, and lansoprazole 30 mg BD) given for 2 weeks at 22 weeks of gestation.	Platelet count increased to 12100 cells/mL after 2 weeks of therapy and remained above 10,000 until delivery. On a postpartum day 1, maternal platelet count was 16100 cells/mL and did not decrease during the next pregnancy without treatment. No maternal perinatal or fetal abnormalities were noted.
Ono et al. [35] (Case 2)	2017	Japan	31/F G1P1	Low platelet count 2.3x10^8 per L at 23 weeks of gestation ( her platelet count at early gestation was normal i.e. 23x10^9/L). The petechial rash was present on lower legs	No established cause of thrombocytopenia. Bone marrow aspiration could not be done due to low platelet. HP IgG antibody and urine test was positive	2.3x10^8 per L	Platelet transfusion and IVIG was given initially that increase her platelet count to 7.2x10^9/L. HP eradication therapy initiated at 24 weeks of gestation	Platelet count increased to 11.7x10^9/L after 2 weeks of eradication therapy and remained stable until delivery. No maternal perinatal or fetal abnormalities
Ono et al. [35] (Case 3)	2017	Japan	32/ F G2P1	Low platelet count i.e. 3.6x10^9/L at 19 weeks of gestation	Platelet count 3.6x10^9/L at 19 weeks of gestation. Bone marrow aspiration refused by the patient. No other identifiable cause of thrombocytopenia. Urea breath test positive	3.6x10^9/L	HP eradication therapy was given at 19 weeks of gestation	Platelet count increased to 10.6x10^9/L after 2 weeks of eradication therapy. The patient had an uncomplicated vaginal delivery. No maternal perinatal or fetal abnormalities.
Ono et al. [35] (Case 4)	2017	Japan	24/F G0P0	Platelet count 8.4x10^9/L at 35 weeks of gestation	Bone marrow aspiration refused by the patient. HP-IgG urine was positive	8.4x10^9/L	HP eradication therapy was given at 36 weeks of gestation	Her platelet count did not recover fully but remained more than 5x10^9/L throughout her pregnancy. She had an uncomplicated vaginal delivery at 39 weeks with no maternal perinatal or fetal abnormalities
Goto et al. [36]	2001	Japan	53/F	Diagnosed case of ITP, treated with prednisone and splenectomy without remission	GI endoscopy showed superficial gastritis. Rapid urease test and the histologic exam revealed H. Pylori. PAIgG 695 ng/ 10^7	24x10^9/L	HP eradication therapy was given	After therapy, platelet count was 134x10^9/L and PAIgG 33ng/10^7

In our literature review, HP-induced ITP was observed in all age groups, with a mean age of 45.7 years (range: 24-78 years). There was some gender preponderance with female predominance just like other autoimmune disorders (male, n=3; female, n=8). The typical presentation was either an incidental diagnosis of low platelet count or some form of mucocutaneous bleeding, such as epistaxis, ecchymosis, bleeding gums, or even hematochezia. Our patients have no underlying identifiable cause of thrombocytopenia other than the HP infection. The diagnosis was established by non-invasive tests like a urea breath test, anti-HP antibodies, or invasive tests in a few cases such as endoscopy and biopsy. It was an interesting observation that for all the cases, the initial low platelet count responded to HP eradication therapy without any relapse.

It is further notable that there were few cases where HP-induced ITP occurred in pregnant patients. The platelet counts were adjusted for dilutional thrombocytopenia in such patients. The non-immunosuppressive treatment, i.e. HP* *eradication therapy in such patients was safe for mother as well as child, as there was no antenatal or perinatal mortality or morbidity found.

## Conclusions

HP infection is an important, yet underrated, cause of secondary thrombocytopenia. The exact pathophysiology of HP-associated ITP is still unclear and further studies are mandated to understand this association as well as determine why there is a variable increase in platelet count in different geographical areas following HP eradication. Every patient with unexplained thrombocytopenia should undergo HP testing, including non-invasive tests, such as urea breath test, anti-HP antibodies, and stool antigen test, followed by invasive tests such as endoscopy and biopsy.* *HP eradication therapy with triple therapy (amoxicillin, clarithromycin, and PPI) should be tried in every ITP case where HP testing is positive or patients aren’t responding to conventional therapies. Our study noted that HP* *eradication therapy is safe in pregnant patients without any antenatal or perinatal mortality or morbidity and/or adverse fetal outcomes.
